# The Numerical Predominance and Large Transcriptome Differences of Neutrophils in Peripheral Blood Together Inevitably Account for a Reported Pulmonary Tuberculosis Signature

**DOI:** 10.1155/2017/5830971

**Published:** 2017-02-06

**Authors:** Kang Wu, Ka-Wing Wong, Wang-Long Deng, Hao Zhang, Jing Li, Douglas B. Lowrie, Xiao-Yong Fan

**Affiliations:** ^1^Shanghai Public Health Clinical Center, Key Laboratory of Medical Molecular Virology of MOE/MOH, Fudan University, 2901 Caolang Road, Shanghai 201508, China; ^2^Shanghai Medical College, Fudan University, 138 Yixueyuan Road, Shanghai 200032, China; ^3^State Key Laboratory of Medical Genomics, Ruijin Hospital Affiliated to Shanghai Jiao Tong University School of Medicine, Shanghai 200025, China; ^4^Department of Pathology, Qiqihar Medical University, Qiqihar 161006, China; ^5^Department of Genetics and Biochemistry, Clemson University, Clemson, SC 29634, USA

## Abstract

Previous transcriptomic analysis revealed a 393-transcript signature (PTBsig), which is dominated by interferon inducible genes, in whole blood of pulmonary tuberculosis (PTB) patients. Comparisons with a limited set of interferon-driven genes among separated monocytes, CD4+ T cells, CD8+ T cells, and neutrophils indicated that the signature is due to changes in neutrophils, the overwhelmingly predominant cell type. By extending the analysis to the entire 393 transcripts of PTBsig and by switching the cell proportions between separated monocytes, CD4+ T cells, CD8+ T cells, and neutrophils, we create putative PTBsig for whole blood (pPTBsig) in which CD4+ or CD8+ T cells or monocytes predominated or in which the cell proportions were unchanged. These putative signatures are then compared to the actual reported PTBsig. We show that, because of their predominance in peripheral blood and their larger transcriptional responses, neutrophils were indeed almost exclusively responsible for PTBsig. We caution that the functional significance of changes in other cell types might escape notice in transcriptome analysis that is based upon whole blood.

## 1. Introduction

Analysis of whole blood without preliminary separation into different components can be an attractive clinical option when considering economy and convenience of investigative and diagnostic procedures. The concept is now being tested in whole blood transcriptome studies in a range of diseases [1] including tuberculosis (TB). In 2010 the first such study in pulmonary TB (PTB) [2] showed that the disease was reflected in whole blood by an interferon- (IFN-) inducible change detected in the expression profile of 393 transcripts (307 genes) and this PTB signature (PTBsig) was extinguished as the disease resolved under effective therapy. The blood cells were also physically separated into monocytes, CD4+ T cells, CD8+ T cells, and neutrophils for transcriptome profiling. Comparisons based on 32 IFN pathway-related transcripts (26 genes) of PTBsig showed PTBsig to be associated with the neutrophils. This analysis, however, did not allow for the differing relative frequencies of different cell types. Neutrophils typically comprise about 50% or more of the leukocytes in whole blood and CD4+, CD8+, and monocyte cells together only account for around 20% or less. Since the transcriptome in whole blood is a direct product of both the magnitude of the changes in gene mRNA levels and the frequency of different blood cells displaying those changes, PTBsig might have been inherently biased towards neutrophil responses. If so, the signature may have little functional relationship to the responses in the majority of TB lesions such as the secondary granulomas where the bacteria reside, but neutrophils are not evident as a major cellular component [3, 4]. To help clarify this issue we here tested more rigorously the degree to which PTBsig was dependent upon the high cell proportion of neutrophils in whole blood and the magnitude of their responses. In contrast to the previous study that was based on comparisons on only 32 IFN pathway-related transcripts in PTBsig, we here used the entire set of 393 transcripts of PTBsig from each of the separated cell populations. We ran analyses based upon switching the cell proportions to create putative PTBsig for whole blood (pPTBsig) in which CD4+ or CD8+ T cells or monocytes predominated or in which the cell proportions were unchanged. These putative signatures were then compared to the actual reported PTBsig. The results indicated that indeed almost in its entirety PTBsig was due to a combination of an overwhelming preponderance of neutrophils and larger magnitude of changes in the gene expression levels of neutrophils.

## 2. Methods

### 2.1. Generation of the Putative Whole Blood Expression Profiles of PTBsig (pPTBsig)

**Figure 1:**
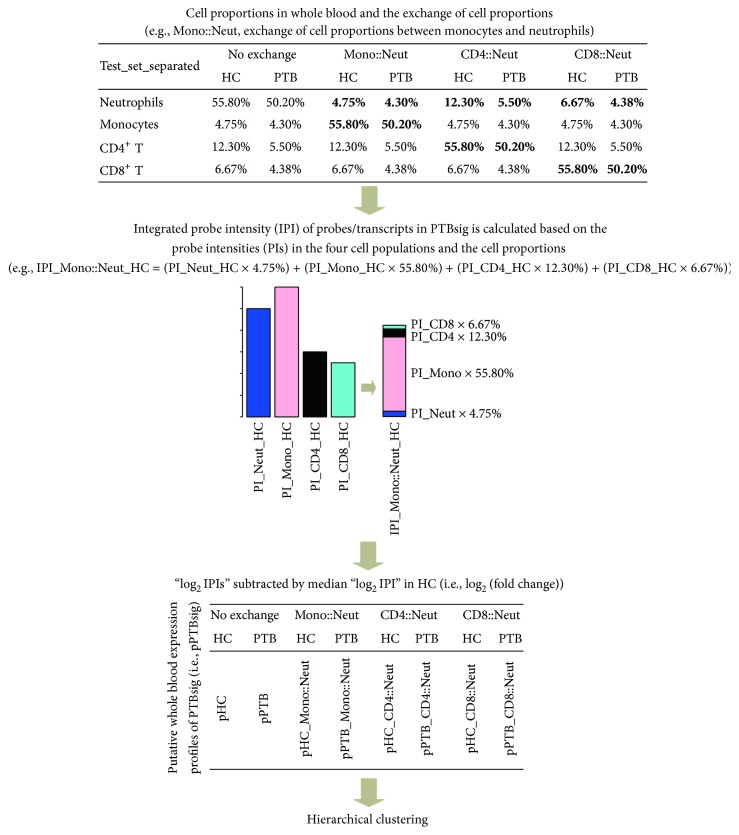
Calculation of putative whole blood PTBsig (pPTBsig). Median cell proportions of the four separated cell populations in HC donors and PTB patients (i.e., neutrophils, monocytes, and CD4+ and CD8+ T cells) were obtained from Figure 3b of the published research [2]. The cell proportions were also artificially exchanged between neutrophils and the other three cell populations (e.g., Mono::Neut, exchange of cell proportions between monocytes and neutrophils). Then the integrated probe intensity (IPI) for each probe/transcript in PTBsig was calculated based on its probe intensity (PI) in the four cell populations and the (exchanged) cell proportions. Next, IPI was log_2_ transformed and subtracted from the median log_2_ IPI in HC donors. The output profiles demonstrated the relative expression levels (log_2_ transformed fold changes) of each probe/transcript in PTB patients compared to HC donors. pHC = pPTBsig based on the four cell populations from HC donors in Test_set_separated with no exchange of cell proportions. pHC_Mono::Neut = pPTBsig based on the four cell populations from HC donors in Test_set_separated with the exchange of cell proportions between neutrophils and monocytes. The other pPTBsig names were derived by similar abbreviation.

To test if the association of PTBsig with neutrophils was simply due to the population dominance of neutrophils, we generated a series of putative whole blood PTBsig (pPTBsig) based on the expression in the separate populations of neutrophils, monocytes, and CD4+ and CD8+ T cells and on different assumed proportions of those cells in the whole blood (Figure 1). The assumed cell proportions were achieved through the exchanges of real cell proportions between neutrophils and the other three cell populations in HC donors or PTB patients (see Figure 1 in detail for the assumed cell proportions). The different pPTBsigs were then compared among themselves or with PTBsig through hierarchical clustering. In detail, median values of the cell proportions of neutrophils, monocytes, and CD4+ and CD8+ T cells were obtained from Figure 3b in Berry et al. [2] (also see upper panel of our Figure 1). These four cell populations together accounted for roughly 80% of the nucleated cells in whole blood of healthy control (HC) donors and 65% in whole blood of PTB patients. The normalized transcriptome array data (GSE19491) was downloaded from NCBI Gene Expression Omnibus. The published PTBsig was defined by 393 transcripts, of which only 32 were used for comparisons between cell populations [2]. We used the probe intensities (PIs) of all 393 transcripts (probes) from the four separated cell populations to calculate pPTBsigs for hypothetical whole blood. The integrated PI (IPI) for each probe was calculated from the summation of the PIs in the four cell populations multiplied by their cell proportions (middle panel of Figure 1). For example, switching the cell proportions of monocytes and neutrophils of the HC donor group, we generated IPI_Mono::Neut_HC, which was calculated as (PI_Neut_HC × 4.75%) + (PI_Mono_HC × 55.80%) + (PI_CD4_HC × 12.30%) + (PI_CD8_HC × 6.67%). IPIs were similarly calculated for the other donor groups, with and without cell proportion exchanges, and then log_2_ transformed and subtracted from the median log_2_ IPIs of the cognate HC donor group. The resultant pPTBsigs gave a representation of log_2_ (fold changes) compared to HC donors under the four conditions (i.e., “no exchange,” “Mono::Neut,” “CD4::Neut,” and “CD8::Neut”) and were used for clustering analysis together with the PTBsigs in Training_set, Test_set, Test_set_separated, and Longitudinal_set as defined in Berry et al. [2]. Cluster 3.0 [5] and TreeView-1.1.6 [6] with correlation (uncentered) were used for unsupervised classification.

### 2.2. Gene Set Enrichment Analysis (GSEA) of PTBsigUp and PTBsigDn in Different Cell Populations

**Figure 2:**
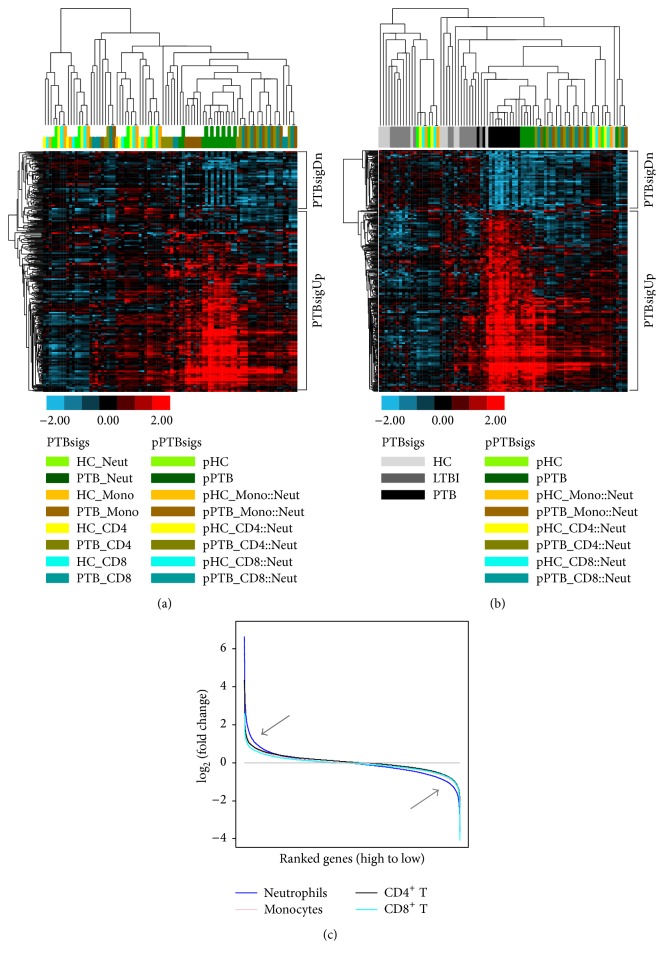
Neutrophils are the main cells responsible for PTBsig. (a) and (b): hierarchical clustering of the pPTBsigs with PTBsigs in Test_set_separated (a) and Training_set (b). HC_Neut = PTBsigs in neutrophils of HC donors. pHC = pPTBsigs based on the four cell populations from HC donors in Test_set_separated with no exchange of cell proportions. pHC_Mono::Neut = pPTBsigs based on the four cell populations from HC donors in Test_set_separated with the exchange of cell proportions between neutrophils and monocytes. The other pPTBsig names were derived by the similar abbreviation. (c) and (Table 1): plots of the relative global responses of the four cell populations in PTB patients (c) and their correlation analysis with PTBsig (Table 1).

The PTBsig that was generated based on the Training_set comparison between PTB patients and HC donors was further grouped into the upregulated subsignature (PTBsigUp, 295 transcripts) and downregulated subsignature (PTBsigDn, 98 transcripts) (Figure 2(b)). To do this we used Linear Models for Microarray Data (LIMMA) [7] to identify the differential expression status in the four separated cell populations between PTB patients and HC donors; then the genes in the separated cell populations of PTB patients were ranked from high to low based on their relative expressions compared to HC donors. GSEA [8] was applied to determine whether PTBsigUp or PTBsigDn as a whole showed statistically significant positive or negative correlation to the ranked genes in the separated cell populations of PTB patients. The reported normalized enrichment score (NES) and false discovery rate (FDR) were used to interpret the GSEA. Positive NES indicated that the gene signature was overrepresented at the top of the ranked genes (indicating the upregulation of a gene signature in general in that cell population), whereas negative NES indicated that the gene signature was overrepresented at the bottom of the ranked genes (indicating the downregulation of a gene signature in general in that cell population). A FDR of 0.05 or less indicated statistical significance of the NES.

## 3. Results

### 3.1. The pPTBsigs with No Exchange of Cell Proportions Were the Closest to the PTBsigs in Neutrophils of PTB Patients

Among the separated cell populations in Test_set_separated, only the PTBsigs in neutrophils of PTB patients (short dark green bar) generally clustered together with the PTBsigs in monocytes of PTB patients (short dark orange bar) (Figure 2(a)). Neither the PTBsigs in neutrophils of PTB patients nor the PTBsigs in monocytes of PTB patients clustered together with other PTBsigs.

Explaining more fully, putative PTBsigs (pPTBsigs) were first calculated based on the gene expression levels and the (assumed) cell proportions of the four separated cell populations (Figure 1); we then tested the relationship between these pPTBsigs and the PTBsigs in each of the four separated cell populations. The pPTBsigs of pPTB (calculated with no exchange of cell proportions, long dark green bar) were usually the closest to the PTBsig_PTB_Neut, being mixed together in the hierarchical clustering tree (Figure 2(a)). Other pPTBsigs from PTB patients with assumed cell proportions (i.e., pPTB_Mono::Neut, pPTB_CD4::Neut, and pPTB_CD8::Neut) generally did not cluster together with the PTBsigs from the separated cell populations of PTB patients. They generally grouped together and were relatively closer to the pPTBsigs of pPTB and PTBsigs of PTB_Neut than to the pPTBsigs of pHC or the PTBsigs from HC donors. PTBsigs and pPTBsigs from HC donors were indistinguishable, being mixed together in the hierarchical tree. Thus the pPTBsigs_pPTB, which were calculated based on the real cell proportions of those four cells populations in PTB patients, inherited most of the expression characteristics of neutrophils of PTB patients.

### 3.2. The pPTBsigs Calculated with No Exchange of Cell Proportions Were the Closest to the Actual Whole Blood PTBsigs in PTB Patients

**Figure 3:**
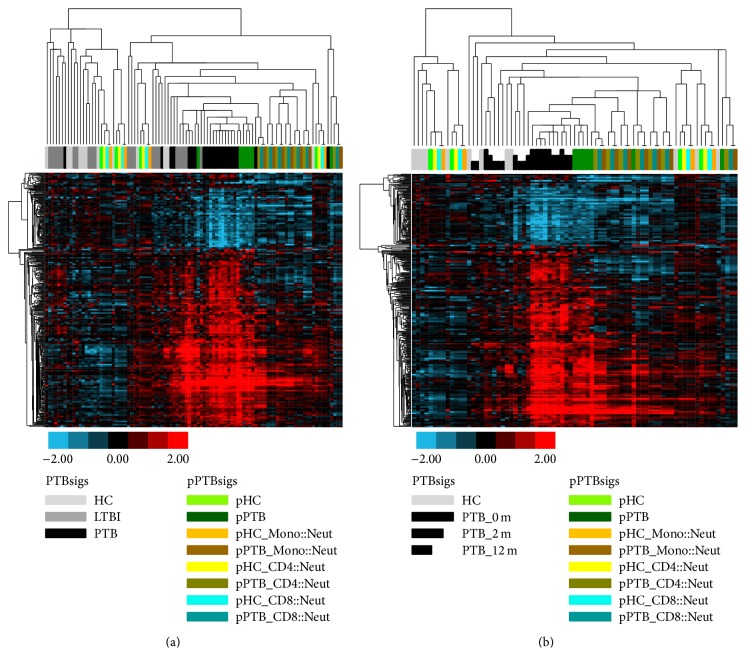
Hierarchical clustering of the pPTBsigs in Test_set (a) and Longitudinal_set (b). pHC = pPTBsigs based on the four cell populations from HC donors in Test_set with no exchange of cell proportions. pHC_Mono::Neut = pPTBsigs based on the four cell populations from HC donors in Test_set with the exchange of cell proportions between neutrophils and monocytes. The other pPTBsig names were derived by the similar abbreviation. PTB_0 m = PTBsigs in whole blood of PTB patients at initiation of drug treatment. PTB_2 m = PTBsigs in whole blood of PTB patients 2 months after drug initiation. PTB_12 m = PTBsigs in whole blood of PTB patients 12 months after drug initiation.

After testing the relationship between the PTBsigs and the pPTBsigs in the four separated cell populations, we then tested the relationship between pPTBsigs and the actual whole blood PTBsigs in HC donors, LTBI donors, and PTB patients. We used the data from Training_set, Test_set, and Longitudinal_set as described in Berry et al. [2]. In Training_set (Figure 2(b)), the PTBsigs in PTB patients were in general different from the PTBsigs in LTBI and HC donors, even though two of the 13 PTBsigs in PTB patients clustered among those in LTBI and HC donors. The PTBsigs in LTBI and HC donors were indistinguishable, being mixed together in the hierarchical tree. This clustering pattern of the PTBsigs in PTB patients and LTBI and HC donors (Figure 2(b)) was consistent with that reported in the original research [2]. The pPTBsigs that were the closest to the PTBsigs in PTB patients were those of pPTB (dark green bar); that is, they were those that were calculated based on the real cell proportions of the four cell populations. Other pPTBsigs, which were calculated based on the exchanges of cell proportions between neutrophils and the other three cell populations, were far away from the PTBsigs in PTB patients. Nevertheless, those pPTBsigs based on the exchanges of cell proportions from PTB patients were closer to the PTBsigs in PTB patients rather than to the PTBsigs in LTBI and HC donors. In contrast, half of the pPTBsigs from HC donors were closer to the PTBsigs in LTBI and HC donors; the other half were closer to the pPTBsigs in PTB patients. A similar clustering pattern was also observed in the Test_set (Figure 3(a)). When considering the Longitudinal_set derived from the clinical therapy of PTB patients, the pPTBsigs of pPTB in general were closer to the PTBsigs in pretherapy PTB patients (PTB_0 m) and early treatment of PTB patients (PTB_2 m) rather than to the PTBsigs at the completion of PTB patient treatment (PTB_12 m) (Figure 3(b)). Thus, the actual whole blood PTBsigs in PTB patients shared most of the expression characteristics of the pPTBsigs being calculated based on the real cell proportions of the four cell populations (neutrophils are dominant cells) in PTB patients and thus inherited most of the expression characteristics of neutrophils of PTB patients.

### 3.3. The Transcriptome Changes in Neutrophils Were Larger and More Numerous Than in the Other Three Cell Populations in PTB Patients and Showed the Strongest Correlations

**Figure 4:**
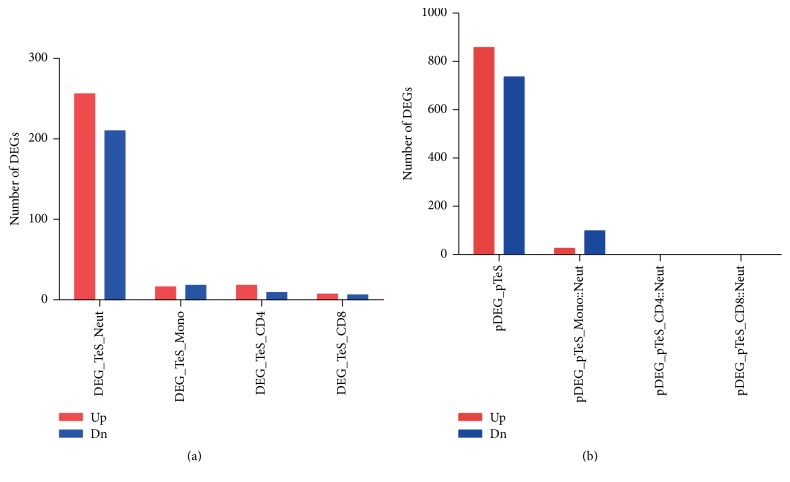
Number of differentially expressed genes (DEGs) in the four separated cell populations (a) and in putative whole blood transcriptome data (b) of PTB patients. In the normalized transcriptome array data GSE19491 (downloaded from NCBI Gene Expression Omnibus), any probe intensity (probe signal) less than 10 was set to 10. Probes with intensity larger than 10 in at least 50% of the 498 samples were selected. The 16,287 probes so selected were used for the subsequent identification of DEGs in the four separated cell populations as well as in the putative whole blood transcriptome data. Linear Models for Microarray Data (LIMMA) were applied for the identification of DEGs [7]. LIMMA used linear models and empirical Bayes methods (the moderated *t*-statistic) in assessing differential gene expression. The criteria for DEGs identification were log_2_ (fold change) ≤ −1 or ≥1 with an adjusted *P* ≤ 0.01 corrected using Benjamini and Hochberg procedure [7]. Putative whole blood transcriptome data were then generated exactly the same as for the generation of putative PTBsig (pPTBsig) (Figure 1), except that in this case the putative whole blood transcriptome data was from 16,287 probes of gene array whereas pPTBsig had only 393 transcripts/probes. The putative whole blood transcriptome data were normalized based on quantiles for the cross-array comparison and then used for identification of DEGs. DEG_TeS_Neut = DEGs in neutrophils of PTB patients compared to neutrophils of HC donors from Test_set_separated; pDEG_pTeS = putative DEGs of putative whole blood of PTB patients compared to putative whole blood of HC donors based on the four cell populations of Test_set_separated with no exchange of cell proportions; pDEG_pTeS_Mono::Neut = putative DEGs of putative whole blood of PTB patients compared to putative whole blood of HC donors based on the four cell populations with the exchange of cell proportions between monocytes and neutrophils. Other abbreviated names were similarly derived.

When the relative expression levels of the four separated cell populations from PTB patients compared to HC donors were ranked from high to low, the genes in the neutrophils of PTB patients were more responsive in transcription than in the other three cell populations, no matter whether the genes were upregulated or downregulated (Figure 2(c)). Likewise, analysis of the whole dataset (48803 probes) revealed more genes being differentially expressed in the neutrophils of PTB patients (i.e., DEG_TeS_Neut, >400) than in the other three cell populations (<50) (Figure 4(a)). Correspondingly, the number of putative DEGs calculated from the whole dataset with no exchange of cell proportions (i.e., pDEG_pTeS) far outnumbered the numbers of DEGs calculated from the whole dataset with the exchanges of cell proportions between neutrophils and the other three cell populations (Figure 4(b)). Additionally, after dividing the 393 PTBsig transcripts into two groups (i.e., PTBsigUp, upregulated in PTB patients compared to HC donors; PTBsigDn, downregulated in PTB patients compared to HC donors), we tested the relationship of PTBsigUp and PTBsigDn to the changes in the four separated cell populations of PTB patients by GSEA. PTBsigUp displayed the highest positive correlation to the neutrophils of PTB patients, and PTBsigDn displayed the lowest negative correlation to the neutrophils of PTB patients (Table 1), indicating the 393 PTBsig transcripts displaying the highest consistent expressions in the neutrophils compared to the other three cells populations of PTB patients. These results confirmed that the neutrophils of PTB patients displayed the highest global response, and the transcripts in PTBsig had the strongest correlation to the neutrophils of PTB patients.

## 4. Discussion

Neutrophils are typically the most abundant leukocytes in peripheral blood, and this remains so in PTB patients. The discovery that a novel whole blood transcriptome profile in PTB patients (PTBsig) was attributable to the changes in gene expression in neutrophils [2] might merely have reflected the numerical predominance of neutrophils. However, the present reanalysis shows that this was not the sole explanation. The neutrophils also had the largest numbers of genes significantly modulated and had larger changes in the level of gene expression, as assessed by probe intensity. The clustering patterns the PTBsigs in HC donors, LTBI donors, and PTB patients were essentially the same as found in Berry et al. [2]; they were not precisely the same because of differing details in the clustering software applications. Recalculating expression profiles after mathematically switching cell proportions did not generate profiles with close similarity to PTBsigs, thereby confirming the association with neutrophils. Nevertheless the pPTBsigs that were generated with monocytes switched for neutrophils (pPTB_Mono::Neut; Figure 2(b)) clustered closer to the pPTBsigs (pPTB) which were generated without switching the cell proportions than did the other switched pPTBsigs (pPTB_CD4::Neut and pPTB_CD8::Neut), suggesting some relatedness between monocytes and neutrophils in their gene expression in PTB patients. However, most of the genes in monocytes of PTB patients that showed changed expression compared to HC donors (14 out of the 16 that were upregulated, 10 out of the 18 that were downregulated) were different from those changed in the other cell types (data not shown), possibly suggesting the existence of a unique monocyte-associated PTB signature, but studies with larger sample sizes would be needed to clarify this since, for example, only 4 HC donors were included in the study [2].

Publication of several TB blood transcriptomic studies followed the one that we analyzed here and further substantiated the prominence of an IFN-inducible response in neutrophils [9–11]. However, there are diverse views on how neutrophils arriving in diseased foci from circulation might contribute to protective immunity or pathology [9, 12]; histologically neutrophils are not usually seen in substantial numbers in and around secondary granulomas but may only be prominent in necrotic and exudative lesions [3, 4]. Furthermore, the signature has not proven to be unique to PTB [10]. Hence, the concept of using peripheral blood to provide transcriptome signatures that are clinically useful across the range of TB status requires further development to reach general fruition [13, 14] and the ideal of having correlates based on proven functional significance of transcriptome signatures is some distance away [15].

## 5. Conclusions

In summary, due to the population predominance of neutrophils in peripheral blood and their higher transcriptional responses, it is inevitable that PTBsig is almost exclusively attributed from neutrophils. It is cautious that functional significance of changes in other cell types of peripheral blood might escape notice in transcriptome analysis that is based upon whole blood.

## Supplementary Material

Supplementary Table 1: List of differently expressed genes in relevant group-group comparisons provided to complement Figure 4. The genes listed all had adjusted *P* = 0.01 and log2 (fold change) = −1 or = 1.

## Figures and Tables

**Table 1:** 

Group comparison^1^	PTBsigUp			PTBsigDn		
	NES^2^	FDR^3^	Correlation^3^	NES^2^	FDR^3^	Correlation^3^
Neut_PTB versus Neut_HC	3.64	0.000	Positive	−1.97	0.000	Negative
Mono_PTB versus Mono_HC	3.53	0.000	Positive	−1.92	0.000	Negative
CD4_PTB versus CD4_HC	2.93	0.000	Positive	−1.82	0.000	Negative
CD8_PTB versus CD8_HC	2.66	0.000	Positive	−1.71	0.000	Negative

^1^Neut_PTB versus Neut_HC = ranked relative expressions of genes of neutrophils in PTB patients compared to neutrophils in HC donors. Similar explanations apply for the other three group comparisons. ^2^(+) NES for positive correlation indicating that signature genes were overrepresented at the higher end of a ranked gene list; (−) NES for negative correlation indicating that signature genes were overrepresented at the lower end of a ranked gene list. ^3^A FDR of 0.05 or lower was regarded as statistically significant for NES (“positive” or “negative”).
